# Xenon inhalation attenuates neuronal injury and prevents epilepsy in febrile seizure Sprague-Dawley pups

**DOI:** 10.3389/fncel.2023.1155303

**Published:** 2023-08-14

**Authors:** Yao Cheng, Yujie Zhai, Yi Yuan, Hao Li, Wenke Zhao, Zhenhai Fan, Ling Zhou, Xue Gao, Yan Zhan, Hongliu Sun

**Affiliations:** ^1^School of Pharmaceutical Sciences, Binzhou Medical University, Yantai, China; ^2^School of Medical Imaging, Binzhou Medical University, Yantai, China; ^3^Department of Neurology, Yantai Affiliated Hospital of Binzhou Medical University, Yantai, China

**Keywords:** febrile seizure, glutamate, xenon, epilepsy, neuronal injury, oxidative stress

## Abstract

**Background:**

Febrile seizures (FS) usually occur in childhood and may cause irreversible neuronal damage, cognitive functional defects, and an increase in the risk of epilepsy later in life. Anti-epileptic drugs (AEDs), currently used to treat FS in children, can relieve seizures. However, their effects in preventing the risk of developing epilepsy in later life are unsatisfactory. Moreover, AEDs may damage child brain development. Here, we evaluated the efficiency of xenon in treating prolonged FS (PFS) and preventing epilepsy in Sprague-Dawley pups.

**Methods:**

Prolonged FS was induced by hyperthermic treatment. After 90 min of PFS, the pups in the xenon treatment group were immediately treated with 70% xenon/21% oxygen/9% nitrogen for 60 min. The levels of glutamate, mitochondrial oxidative stress, mitophagy, and neuronal injury, seizures, learning, and memory functions were measured at specific time points.

**Results:**

Neonatal period PFS led to spontaneous seizure, learning and memory dysfunction, accompanied by increased levels of glutamate, mitochondrial oxidative stress, mitophagy, and neuronal injury. Xenon treatment alleviated the changes caused by PFS and reduced the risk of PFS developing into epilepsy later.

**Conclusion:**

Our results suggest that xenon inhalation could be a potential therapeutic strategy to attenuate neuronal injury and prevent epilepsy in patients with FS.

## 1. Introduction

Febrile seizures (FS) are the most common type of seizure induced by hyperpyrexia (38°C or higher) and may lead to varying degrees of negative effects in the central nervous system (Graves et al., 2012; Patel and Vidaurre, 2013). FS often occur between 6 months and 5 years of age and affect 2–5% of children (Patel and Vidaurre, 2013). Clinical data suggest that a history of FS in childhood is associated with approximately 13% of epilepsy cases (Moreno de Flagge, 2013) and 37.1% of sudden unexplained deaths in childhood (Hesdorffer et al., 2015). Based on their clinical manifestations, FS are classified into simple and complex (Mewasingh, 2014). Complex FS last longer than 15 min (Graves et al., 2012) and exhibit recurrent seizure characteristics (Camfield and Camfield, 2015; Whelan et al., 2017). Up to 40% of complex FS patients with prolonged FS (PFS) develop temporal lobe epilepsy (TLE) (McClelland et al., 2011). Simultaneously, PFS can reorganize functional circuits and change molecular levels in the brain, which can lead to epilepsy in later life (Gibbs et al., 2011).

According to a previous study, glutamate-induced excitotoxicity is closely related with the excessive production of free radicals, which can change the composition of sucrose, lipids, and nucleic acids (DNA or RNA), and thereby, destroys cell function (Elmas et al., 2017). The brain is sensitive to oxidative free radicals and is one of the most vulnerable organs to oxidation (Nazıroglu, 2009; El-Masry et al., 2018). Furthermore, increased nitric oxide (NO) synthases, heme oxygenase-1, and plasma NO formation have been detected in recurrent FS (Yang and Qin, 2004). Besides, El-Masry et al. (2018) found that levels of malondialdehyde and NO were significantly increased, whereas that of superoxide dismutase was decreased in patients with FS. These studies indicate that excitotoxicity-related oxidative stress may play a key role in children with FS (Feng et al., 2016).

Under clinical settings, anti-epileptic drugs (AEDs), such as phenytoin, valproic acid, and phenobarbital, are commonly used for treating FS (Graves et al., 2012). However, while AEDs can alleviate seizure symptoms, they rarely exhibit neuroprotective effects or reduce the risk of future epilepsy (Paul et al., 2015). Furthermore, AEDs may harm cognitive functional development in newborns (Moavero et al., 2017). Moreover, drug resistance to AEDs is a serious problem during therapy, especially in TLE where drug resistance exceeds 50% (Wang et al., 2019). Therefore, safer and more effective treatments are crucial to alleviate seizures and neuronal injury and decrease the risk of FS developing into epilepsy.

Xenon, a colorless and odorless monoatomic noble gas, was discovered in 1898 and first used as an anesthetic in 1951 (Korsunsky, 2015). Xenon is non-flammable, non-explosive, non-toxic, and non-teratogenic (Korsunsky, 2015). Moreover, xenon cannot be metabolized and is discharged through the lungs rather than the liver or kidney, rendering it the preferred treatment for patients with impaired liver and kidney function (Maze and Laitio, 2020). A recent phase II clinical trial demonstrated its safety in patients with cardiovascular diseases (Maze and Laitio, 2020).

Furthermore, the neuroprotective effects of xenon are believed to be significant (Wilhelm et al., 2002). Our previous studies confirmed that xenon mixture treatment significantly alleviates the severity of kainic acid-induced status epilepticus and hypoxia-induced newborn seizures and improves learning and memory defects in hypoxia-induced C57BL neonatal mice (Zhang et al., 2019a,^2020^). Furthermore, xenon inhalation enhances neuronal protection by decreasing mitochondrial oxidative stress (Zhang et al., 2020). More importantly, as a crucial risk factor, neural injury plays a significant role in the development of epilepsy. Therefore, a reduced risk of developing epilepsy in children with serious FS may possibly be achieved by early neuroprotection using xenon treatment.

Here, combined with the strong roles of xenon in neuroprotective properties, we hypothesized that xenon treatment may benefit PFS. To test this hypothesis, we designed an experiment to evaluate the role of xenon inhalation in PFS pup rats to seek a safer and more effective therapeutic strategy for FS.

## 2. Materials and methods

### 2.1. Animals and experimental groups

Sprague-Dawley (SD) rats (2-month-old, Jinan Pengyue Experimental Animal Breeding Co., Ltd., China; No. SCXK 20190003) were used in this study. One male rat was raised with two female rats in one cage until the females became pregnant. All animals were provided with food and water *ad libitum*.

**FIGURE 1:**
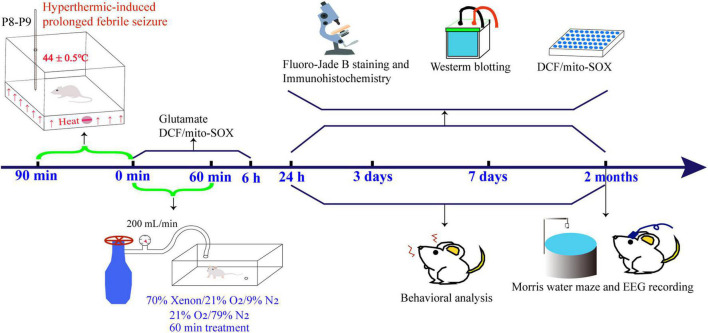
Experimental procedure specifics. EEG, electroencephalography; DCF, 2’,7’-dichloro-dihydro-fluorescein.

Following rigorous experimental principles, the pups were grouped randomly into two groups. The pups in one group were used for experiments in their infancy (pup-group), and the pups in the other group were used for experiments in their adulthood (adult rat group). The pup group was further divided into control (*n* = 42), PFS (*n* = 46), PFS with xenon treatment (xenon; *n* = 29), and PFS without xenon treatment (without xenon; *n* = 29) groups. Similarly, the adult rat group was divided into control (Female, *n* = 16; Male, *n* = 16), PFS (Female, *n* = 16; Male, *n* = 20), xenon (*n* = 32), and without xenon (*n* = 32) groups. The experimental design and details are presented in Figure 1.

### 2.2. Hyperthermic-induced PFS

The day of birth of the pups was defined as P0 (Chen et al., 2016). Pups with weights ranging 15–18 g in each cage were selected for PFS induction at P8–P9 (Baram et al., 1997; Chen et al., 2016; Feng et al., 2016). The pups were placed in a thermostatic chamber (44 ± 0.5°C in homoiothermy) for prolonged hyperthermic treatment (Baram et al., 1997; Chen et al., 2016; Feng et al., 2016). According to previous studies (Toth et al., 1998; Chen et al., 2016), seizures include limb spasms and lateral body flexion with loss of control, followed by “swimming” motions at approximately 15 min after the start of hyperthermia treatment. When seizures were evoked, the pups were placed on a cool surface for 2 min (Baram et al., 1997; Chen et al., 2016). After cooling, the pups were placed in a thermostatic chamber for the next seizure. Typically, it took approximately 13–15 min from the time rats were placed back into the chamber to the second seizure. When the second seizure occurred, the rats were permitted to seizure persistently for 10 min in the thermostatic chamber and subsequently taken out onto the cool surface for 2 min. The third and fourth seizures were similar to the second. The pups had a total of four seizures and the persistent seizure time was approximately 30 min. After the heat treatment, the animals were checked for burns and placed back in the original cage. A total of 283 pups were used in our experiments. Three pups without seizures within 55 min were excluded (Feng et al., 2016). Two pups died owing to serious seizures.

### 2.3. Xenon treatment

After 90 min of hyperthermic treatment, the pups were randomly divided into two groups: with and without xenon (control). The pups in the xenon treatment group were immediately transferred to 70% xenon/21% oxygen/9% nitrogen (50 L, DaTe Special Gas Ltd., China) for 60 min (Zhang et al., 2019a,^2020^). The pups in the control group were treated with 21% oxygen/79% nitrogen for 60 min (50 L; Rulin Gas Ltd., China). The gas mixture was delivered through a flow regulator gate at 200 mL/min (DaTe Special Gas, Ltd.) based on the method used in our previous studies (Zhang et al., 2019a,^2020^). After treatment, the animals were returned to their original cages. All pups were awake during xenon treatment. The pups were separated according to sex after weaning on Day 21.

### 2.4. Behavior and electroencephalographic (EEG) testing

The seizure behaviors of rats were observed at 24 h, 3 days, 7 days, and 2 months. The seizure behaviors were recorded continuously in a video. More precisely, at 2 months, the rats were divided into male and female groups and their behaviors were consistently recorded by video for 3 days. Subsequently, the frequency and duration of seizures over the 3 days were recorded and the daily average was calculated. The 24 h, 3-day, and 7-day seizure behaviors of rats were recorded on video for 24 h. The stages of seizure were evaluated using Racine’s scale (Racine, 1972), and the seizures were divided into five grades: Grade I: facial twitch, blink, freeze; Grade II: rhythmic shaking of the head; Grade III: forelimb spasm; Grade IV: intermittent general ankylotic spasm; and Grade V: persistent general ankylotic spasm. Besides, EEG recording and analysis were performed 2 months after PFS. At 53 days after initial PFS, the rats were anaesthetized with pentobarbital sodium (50 mg/kg, intraperitoneally, CAS, 57–33-0, Xiya Reagent, China), and stainless steel electrodes (0.5 mm tips uncoated; A.M. Systems, USA) were implanted into the left cortex (Zhang et al., 2019b) using a stereotactic apparatus (Anhui Zheng Hua Biological Instrument Equipment Co., Ltd., China). In accordance with brain atlases (the third section), the electrodes were implanted [anteroposterior (AP), -3.2 mm; mediolateral (ML), -3.0 mm; dorsoventral (DV), -1.8 mm] for EEG recording. Subsequently, 7 days after surgery, the EEG of each animal was continuously recorded for 3 days using a Powerlab device (1–50 Hz, AD Instruments, Australia). The standard seizure for EEG was determined as a detection with persistent spiny shapes for at least 3 s and with an amplitude twice that of the background rhythm (Rakhade et al., 2011).

### 2.5. Morris water maze testing

Consistent with the method using in a previous study (Zhang et al., 2019a), learning and memory function were determined on day 60 using the Morris water maze (ZS-001, Beijing Zhongshi Di Chuang Technology Development Co., Ltd., China). The experiment included place navigational and space-exploring tests. The rats were placed in any quadrant for free training for 2 min. If the rats did not find the platform within 60 s, the tester induced them to find it and made the rats stay on it for 10 s. On day 5, the platform was removed for space exploration testing and the rats were placed in the water farthest from the position of the platform and allowed to search for 60 s. Finally, the learning and memory function of the rats was evaluated by the frequency of crossing the target quadrant, time spent in the target and opposite quadrants, and latency of getting to the platform (Netto et al., 1993).

### 2.6. Western blotting

The detection time points were 24 h, 3 days, and 2 months after PFS (*n* = 4/group). Following the procedures described in the previous study (Cheng et al., 2021), the animals were narcotized with pentobarbital sodium (50 mg/kg, i.p.), and the cortex and hippocampus were quickly separated on ice and weighed. Next, a mixture (10 μL/mg) of RIPA lysis buffer (MA0151, Dalian Meilun Biotechnology, China) and protease inhibitor (phenylmethanesulfonyl fluoride, PMSF, ST506, Beyotime, China) in a ratio of 100:1 (RIPA lysis: PMSF) was added and sonicated to break the cells using an ultrasonic homogenizer (SCIENTZ-650E, Ningbo Scientz Biotechnology, China). The proteins were separated using 12% polyacrylamide gel (P0012A; Beyotime, China). The target protein was transferred onto a polyvinylidene fluoride membrane (220 mA, 60 min) and blocked using 5% skim milk. Images were acquired using an Odyssey biomolecular imager (LI-COR Biosciences, USA). The gray values of all the target membranes were normalized to GAPDH.

### 2.7. Fluoro-Jade B (FJB) staining

As a conventional label for neuronal injury (Saganová et al., 2006), FJB staining was performed after PFS (24 h, 3 days, and 2 months; *n* = 4/timepoint/group). The animals were anaesthetized with pentobarbital sodium (50 mg/kg, i.p.), and 250 mL of 0.9% saline was perfused, followed by 250 mL of 4% paraformaldehyde (PAF, PH 7.2–7.4). The brains were then harvested and treated with 4% PAF for 24 h. After gradient dehydration, each frozen sample was cut into 12-μm sections (CM3050s, Leica, Germany) (Cheng et al., 2021). The prepared slices were dried for 30 min at 50°C, bathed in an 80% ethanol solution with 1% NaOH for 5 min and 70% ethanol solution for 2 min, and then placed into deionized water for 2 min. The slices were then placed in 0.06% potassium permanganate solution on a shaking table for 15 min to obtain a clear background, followed by a wash in distilled water for 2 min. Next, the brain sections were incubated for 30 min in the dark with 0.0004% FJB staining working fluid (AG310-30MG, EMD Millipore, USA). Subsequently, images were captured under a fluorescence microscope (Olympus, Japan).

### 2.8. Immunohistochemistry

At 24 h, 3 days, and 2 months, the immunofluorescence intensity of the target proteins was detected (*n* = 4/group). After three washes with 0.01 M phosphate buffered saline (PBS), the sections were blocked with 10% bovine serum albumin (MB4219-2, Dalian Meilun Biotechnology, China) for 1.5 h. Then, the primary antibodies, LC3B (rabbit monoclonal antibody; 1:300; ab48394; Abcam, UK), and translocase of outer mitochondrial membrane 20, (TOMM20, mouse monoclonal antibody; 1:200; ab56783; Abcam, UK) were added to each brain section (50 μL/section) at 37°C for 1 h and then incubated at 4°C for overnight. The sections were washed and incubated with the FITC conjugated goat anti-mouse IgG antibody (1:200; ab20003ss; Absin Bioscience; China) or Cy3-labeled goat anti-rabbit IgG (1:200; A0516; Beyotime, China) for 1.5 h at 37°C in the dark. Next, 2-(4-Amidinophenyl)-6-indolecarbamidine dihydrochloride (DAPI, C1005; Beyotime, China; 50 μL/section) was added to each section for 15 min in the dark and then washed and covered with coverslips. Finally, the images were observed using a fluorescence microscope (Olympus, Japan). The fluorescence intensity and Pearson’s correlation and overlap coefficient were analyzed using Image J 1.8 V (National Institutes of Health, Bethesda, USA).

### 2.9. Reactive oxygen species (ROS) detection

At 0 min, 60 min (immediately after xenon treatment), 6 h, 24 h, 3 days, and 2 months after PFS, the levels of ROS in each group (*n* = 4/group) were detected using an ROS assay kit (S0033; Beyotime, China). After anesthesia, the hippocampus and cortex were separated on ice, and 0.01 M PBS (10 μL/mg) was added to each sample. The tissues were cut on ice and filtered using a 200-mesh screen to make single-cell suspensions. Then, 500 μL of 2’, 7’-dichloro-dihydro-fluorescein diacetate (DCFH-DA; S0033S, Beyotime, China) working liquid (DCFH-DA diluted with a serum-free medium at a ratio of 1:1,000 to 10 μM/L) was added to 250 μL of single cell suspension of each sample and incubated for 40 min without light. Subsequently, the cell suspension was centrifuged at 2,000 rpm and the supernatant was discarded. Centrifugation was performed thrice. The fluorescence intensity was detected at an emission wavelength of 525 nm and an excitation wavelength of 488 nm using a microplate reader (Synergy H1; BioTek Instruments, Inc., USA).

### 2.10. Mitochondrial oxidative stress assessment

Similar to the detection of ROS, the levels of mitochondrial ROS were evaluated at 0 min, 60 min (immediately after xenon treatment), 6 h, 24 h, 3 days, and 2 months after PFS (*n* = 4/group). After the preparation of the single-cell suspension, mitochondrial superoxide (Mito-SOX™) solution (M36008, Thermo Fisher, USA) was added to the tissue (5 μM/mL diluted in DMSO) for 10 min without light. After three centrifugal washes with 0.01 M PBS (5 min each time), the mean fluorescence intensity was detected using flow cytometry (BD Biosciences, USA) and a microplate reader (Synergy H1) at an emission wavelength of 580 nm and excitation wavelength of 510 nm, respectively.

### 2.11. Detection of glutamate concentration

At P8–P9, the concentration of glutamate was detected using high-performance liquid chromatography (HPLC, Agilent Technologies, USA) equipped with a fluorescence detector after PFS treatment (*n* = 5/group). The pups were decapitated, and the hippocampus and cortex were separated on ice immediately. We added 50% methyl alcohol/H_2_O (methyl alcohol: H_2_O = 1:1) according to the ratio 19 mL/g. The brain samples were subsequently broken using an ultrasonic homogenizer (SCIENTZ-650E, Ningbo Scientz Biotechnology, China) and centrifuged at 14,000 rpm and 4°C for 10 min to collect the supernatant (Oreiro-García et al., 2005; Fiechter et al., 2013). Next, the standard glutamate (SG8540, Solarbio, China) was diluted in accordance with the concentration gradient (0.01 mmol/L, 0.025 mmol/L, 0.05 mmol/L, 0.1 mmol/L, 0.2 mmol/L) for a standard curve (*y* = 388.6x + 1.1541, *R*^2^ = 0.9997). The diluted samples were added to sodium borate buffer (S885293, Macklin, China) and AQC (A131410, Alladin, China) according to a ratio of 10:70:20 (Chen et al., 2000). After the derivatization reaction at 55°C for 10 min, 400 μL of pure water was added, and 10 μL of each sample was analyzed at 246 nm (excitation wavelength) and 299 nm (emission wavelength). The samples were analyzed using a Supersil ODS2 C_18_ column (200 mm × 4.6 mm, 5 μm, Elite Analytical Instrument Co., Ltd., China) at 30°C, the mobile phase consisted of eluent A containing 90 mM sodium acetate, 93% ultrapure and 7% acetonitrile (pH = 5.3) and eluent B containing methanol-acetonitrile-water (20:60:20). The samples were separated by gradient eluent at a flow rate of 1 mL/min. The gradient separation procedure was set as follows: 0–5 min, 2% B; 6–15 min, 10% B; 16–27 min, 0% B; 28–47 min, 100% B. Finally, the data were analyzed by Origin 9.0 and prism 9.0.

### 2.12. Ethics statement

All animal experiments complied with the ARRIVE guidelines, animal ethics clause of the Binzhou Medical University Experimental Animals Committee (approval no. 2020002), and National Institutes of Health guidelines for the care and use of experimental animals (National Institutes of Health Publication No. 80-23, 1996 Revision). All efforts were made to reduce the pain experienced by the animals.

### 2.13. Statistical analyses

The sample size was estimated using a balanced one way analysis of variance (ANOVA) based on the pre-experimental data. Statistical tests were justified and the data met normal distribution and variance homogeneity. The data are presented as the mean ± SEM. SPSS (version 25.0; IBM, USA) was used to analyze the statistical data. The latency to platform of our experimental data was analyzed by using two-way ANOVA. One categorical independent variable between three or more groups was analyzed by one-way ANOVA, and unpaired *t*-test was used for analyses between two groups. Fisher’s exact test was used to compare the number of rats with spontaneous seizures between the PFS and xenon treatment groups. Statistical significance was set at *P* < 0.05.

## 3. Results

### 3.1. Special explanation

Based on our experimental design, rats treated with PFS were divided according to sex; however, no significant difference was observed between the male and female groups. Therefore, we combined the data of these two groups (male and female) to assess the role of xenon treatment.

### 3.2. Neonatal PFS led to spontaneous seizures in adulthood

**FIGURE 2:**
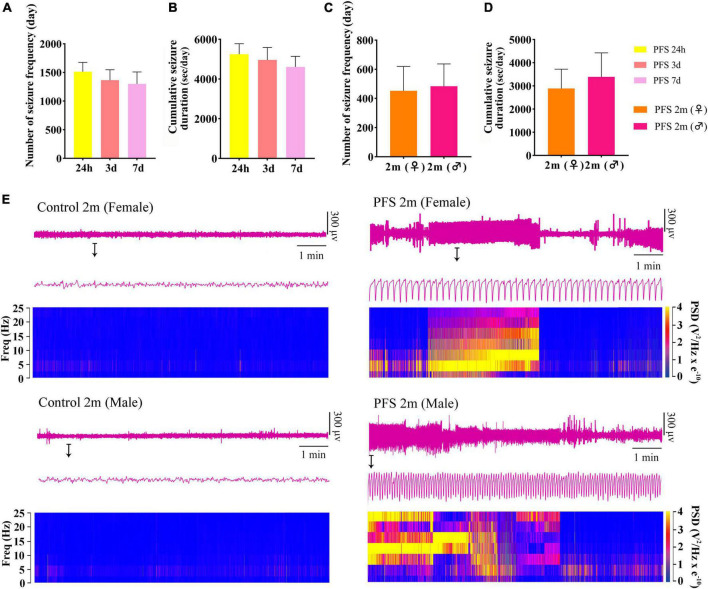
Neonatal PFS led to spontaneous seizures in adulthood. **(A)** Number and **(B)** cumulative duration of behavioral seizures at 24 h (*n* = 20), 3 days (*n* = 18), and 7 days (*n* = 18). **(C)** Number of seizures and **(D)** cumulative seizure duration at 2 months (female, *n* = 16; male, *n* = 20). **(E)** Representative EEG and PSD analysis between control and PFS groups at 2 months. Data are the mean ± SEM. EEG, electroencephalography; PFS, prolonged febrile seizure; PSD, power spectral density; ♂, male; ♀, female.

Seizures were observed at 24 h, 3 days, 7 days, and 2 months after PFS treatment. The number of seizures and cumulative seizure duration at 24 h (*n* = 20), 3 days (*n* = 18), and 7 days (*n* = 18) in the pup group are presented in Figures 2A, B. The number and cumulative duration of behavioral seizures in the adult group are shown in Figures 2C, D. In addition, the seizures of EEGs at 2 months were measured (Supplementary Figures 1A, B). Representative EEGs and power spectral density (PSD) at 2 months are presented in Figure 2E. These results indicate that rats that suffered PFS during neonatal period were more likely to develop spontaneous seizures in adulthood.

### 3.3. Neonatal PFS led to learning and memory dysfunction in adults

**FIGURE 3:**
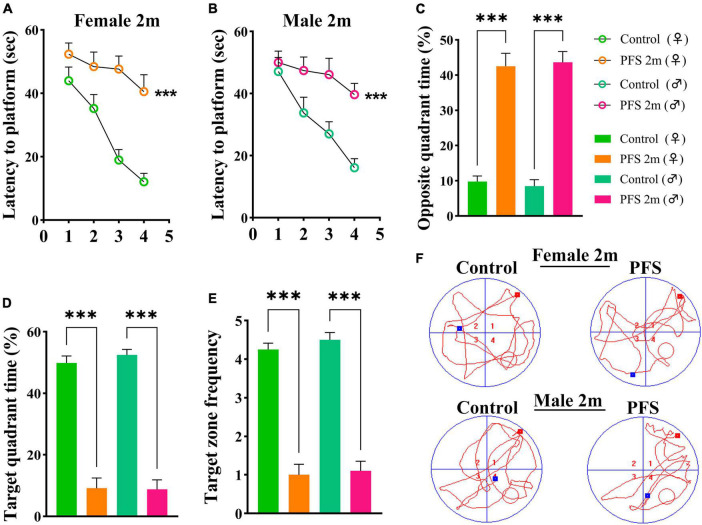
Neanatal PFS led to learning and memory dysfunction in adults. **(A,B)** Latency to platform, **(C)** opposite quadrant time, **(D)** target quadrant time, **(E)** target zone frequency at 2 months. **(F)** Representative trajectory diagrams of the Morris water maze in each group. Female, *n* = 16; male, *n* = 20. ***P < 0.001 vs. control groups, respectively. ♂, male; ♀, female; PFS, prolonged febrile seizure.

The Morris water maze test was used to evaluate learning and memory function 2 months after hyperpyretic treatment (female, *n* = 16; male, *n* = 20). In the PFS group, the latency to the platform (Figures 3A, B) and the percent of opposite quadrant time (female, 42.5 ± 3.7; male, 43.7 ± 3.0, Figure 3C) were significantly higher (*P* < 0.001 in both males and females) than those in the control group (female, 9.8 ± 1.5; male, 8.5 ± 1.8). In contrast, the percent of target quadrant time (female, 9.1 ± 3.3 vs. 49.9 ± 2.3; male, 8.7 ± 3.1 vs. 52.5 ± 1.8; Figure 3D) and target zone frequency (female, 1.0 ± 0.3 vs. 4.3 ± 0.2; male, 1.1 ± 0.3 vs. 4.5 ± 0.2, Figure 3E) were significantly lower in the PFS group than in the control group. Representative track diagrams for each group are shown in Figure 3F. These results indicate that PFS in neonatal period can lead to learning and memory defects in adulthood.

### 3.4. PFS in neonatal period elevated the levels of glutamate and oxidative stress

**FIGURE 4:**
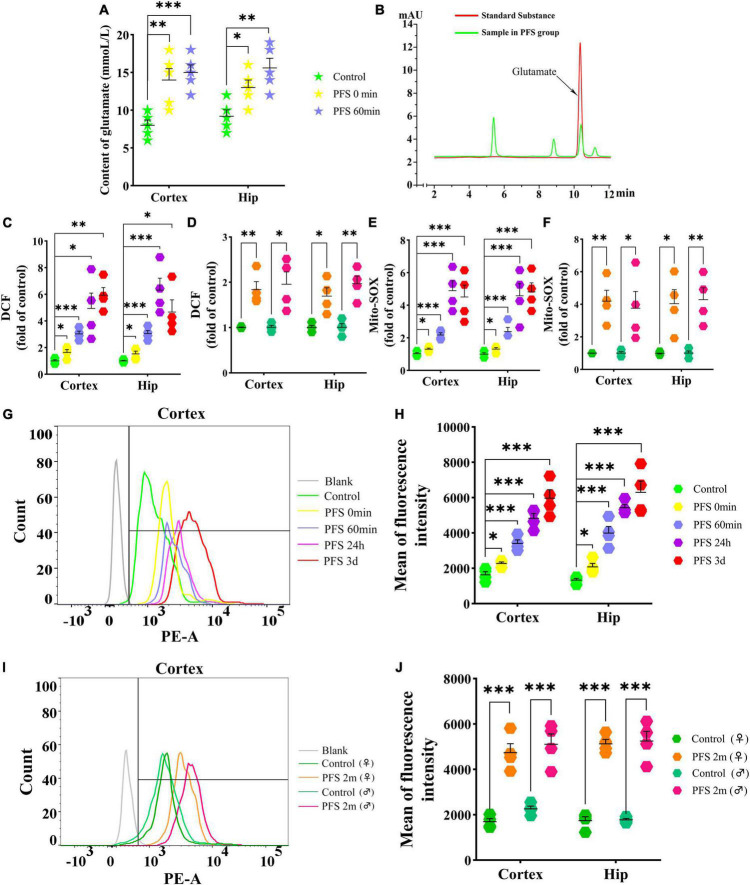
PFS in neonatal period elevated the levels of glutamate and oxidative stress. Levels of glutamate **(A,B)** and DCF **(C,D)** in the cortex and hippocampus after PFS (*n* = 4/group). Levels of mito-SOX detected by fluorescence microplate reader **(E,F)** and flow cytometry **(G–J)** (*n* = 4/group). ***P < 0.001, **P < 0.01, and *P < 0.05 vs. the control group. ♂, male; ♀, female; PFS, prolonged febrile seizure; Hip, hippocampus; DCF, 2’,7’-dichloro-dihydro-fluorescein.

The concentration of glutamate was detected using HPLC after PFS. The results show an evident increase in glutamate levels due to PFS treatment (0 min, cortex, *P* = 0.007, hippocampus, *P* = 0.020; 60 min, cortex, *P* < 0.001, hippocampus, *P* = 0.003; Figures 4A, B). Moreover, the ROS levels were significantly increased in the pups of PFS groups (Figure 4C) and adults (Figure 4D) than in those of the respective control groups. Mito-SOX levels demonstrated a similar increase in PFS groups of both pups and adults (Figures 4E–J). Representative figures of the cortex are presented in Figures 4G, I.

### 3.5. Mitophagy levels increased in rats experiencing neonatal PFS

**FIGURE 5:**
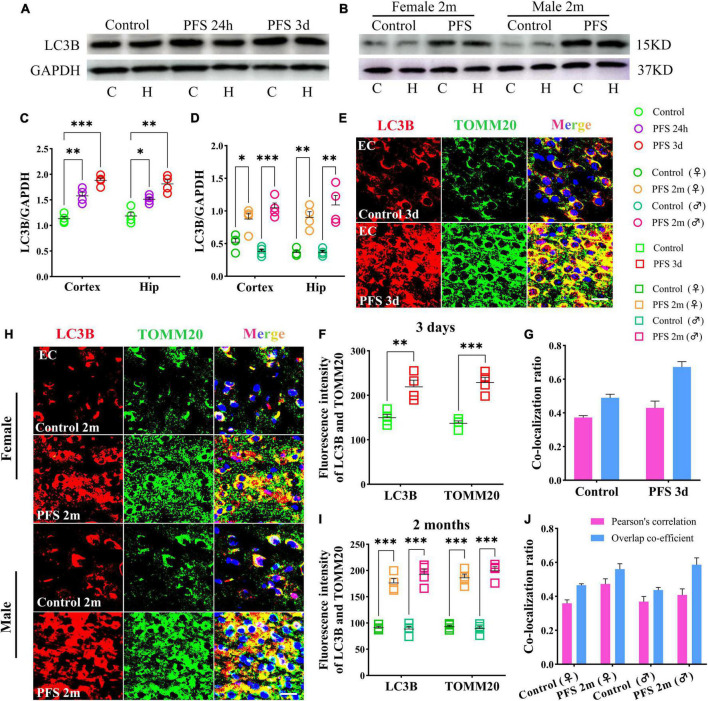
Mitophagy levels increased in rats experiencing neonatal PFS. **(A,B)** Levels of LC3B in the cortex and hippocampus at 24 h, 3 days, and 2 months (*n* = 4/group). **(C,D)** Normalized intensity level of LC3B related to GAPDH. **(E,H)** Immunohistochemistry staining of LC3B (red) and TOMM20 (green) in the EC region at 3 days and 2 months. Bar = 30 μm. DAPI, blue. **(F,I)** Mean fluorescence intensity analysis (*n* = 4/group). **(G,J)** Co-localization ratio of LC3B and TOMM20. ***P < 0.001, **P < 0.01, and *P < 0.05 vs. control groups. C, cortex; EC, entorhinal cortex; Hip, hippocampus; LC3B, microtubule-associated protein light chain 3B; PFS, prolonged febrile seizure; TOMM20, translocase of outer mitochondrial membrane 20; ♂, male; ♀, female.

The autophagy-related protein LC3B was detected at 24 h, 3 days, and 2 months by western blotting (*n* = 4/group). The level of LC3B in early neonatal period is higher than that in adulthood (*n* = 4/group, cortex: *P* = 0.011; hippocampus: *P* = 0.004; Supplementary Figures 2A, B). Therefore, changes in LC3B in both children and adults were compared. The results of western blotting revealed increased levels of LC3B in PFS rats not only in the pup group (cortex: 24 h, *P* = 0.002; 3 days, *P* < 0.001; hippocampus: 24 h, *P* = 0.011; 3 days, *p* = 0.001; Figures 5A, C) but also in the adult group (cortex: female, *P* = 0.016; male, *P* < 0.001; hippocampus: female, *P* = 0.001; male, *P* = 0.003; Figures 5B, D).

In concordance, the results of immunohistochemistry showed a higher mean fluorescence intensity of LC3B and TOMM20 in the PFS group than in the control group of pups (e.g., entorhinal cortex [EC], 3 days, LC3B, *P* = 0.006; TOMM20, *P* < 0.001; Figures 5E, F). The positive signals of LC3B and TOMM20 overlapped (Pearson’s correlation: 0.32 ± 0.42; overlap co-efficient: 0.69 ± 0.75; Figure 5G). Similar increases were observed in the adult PFS group at 2 months (LC3B: female, *P* < 0.001; male, *P* < 0.001; TOMM20: female, *P* < 0.001; male, *P* < 0.001; Figures 5H, I). The overlap coefficient (female, 0.48 ± 0.53; male, 0.66 ± 0.48) and Pearson’s correlation coefficient (female, 0.51 ± 0.42; male, 0.31 ± 0.48) of the PFS group are presented at Figure 5J. Our results suggest that PFS can lead to elevated levels of autophagy, which mainly consists of mitophagy.

### 3.6. PFS led to neuronal injury

**FIGURE 6:**
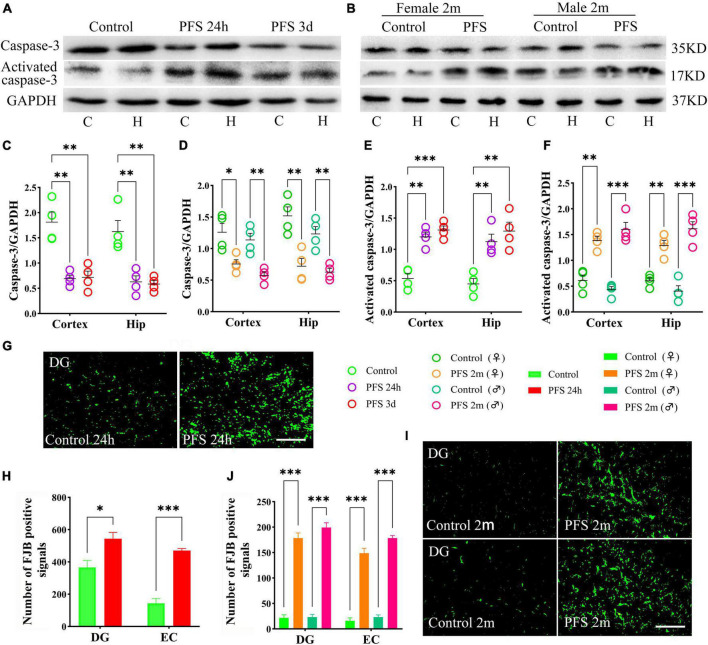
PFS led to neuronal injury. **(A,B)** Apoptosis related proteins of caspase-3 and activated caspase-3 were detected at 24 h, 3 days, and 2 months (*n* = 4/group). Normalized gray value of caspase-3 **(C,D)** and activated caspase-3 **(E,F)**. **(G,I)** Representative results of FJB staining for the DG region at 24 h and 2 months (*n* = 4/group, bar = 50 μm). **(H,J)** Analysis of FJB positive signals. ***P < 0.001, **P < 0.01, *P < 0.05 vs. the control groups. C, cortex; DG, dentate gyrus; EC, entorhinal cortex; FJB, Fluoro-jade B; Hip/H, hippocampus; PFS, prolonged febrile seizure; ♂, male; ♀, female.

The levels of the apoptosis-related proteins detected by western blotting at 24 h, 3 days, and 2 months (*n* = 4/group) revealed decreased levels of caspase-3 (24 h, cortex, *P* = 0.002; hippocampus, *P* = 0.007; 3 days, cortex, *P* = 0.003; hippocampus, *P* = 0.004; and 2 months, cortex: female, *P* = 0.018; male, *P* = 0.003; hippocampus: female, *P* = 0.006; male, *P* = 0.003; Figures 6A–D) and increased levels of activated caspase-3 (Figures 6A, B, E, F) in PFS groups compared with those in the control groups.

Furthermore, more FJB-positive signals were observed in the PFS groups of both pups (24 h, DG: *P* = 0.023; EC: *P* < 0.001; Figures 6G, H) and adults (2 months, DG: female, *P* < 0.001; male, *P* < 0.001; EC: females, *P* < 0.001; males, *P* < 0.001; Figures 6I, J) compared with that in the respective control groups.

### 3.7. Xenon treatment attenuated seizure severity and learning and memory defects caused by PFS

**FIGURE 7:**
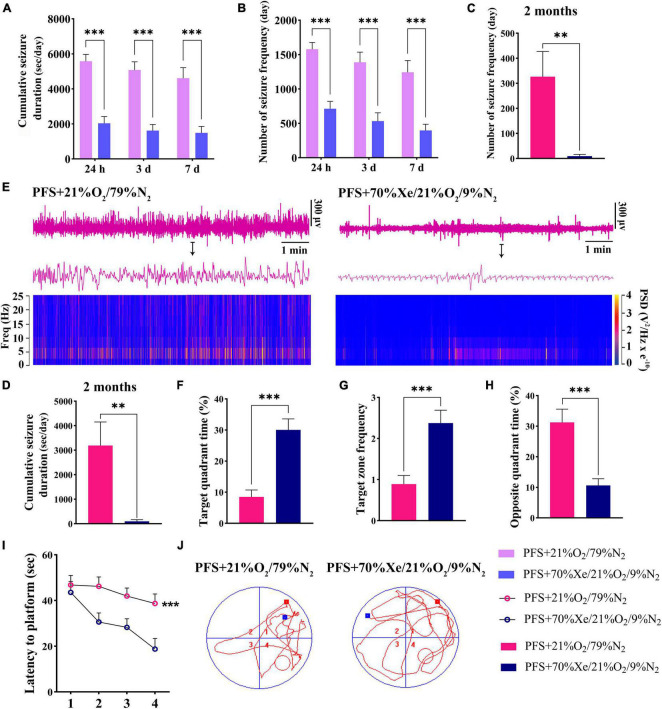
Xenon treatment attenuated the severity of seizures and defects in learning and memory caused by PFS. **(A–D)** Cumulative duration and frequency of behavioral seizures in each group (24 h, 3 days, 7 days; *n* = 16/group/timepoint; 2 months; PFS group, *n* = 18, xenon group, *n* = 16). **(E)** Representative EEG and PSD analyses at 2 months. The statistical data of target quadrant time **(F)**, target zone frequency **(G)**, opposite quadrant time **(H)**, and latency to platform **(I)** suggest that the learning and memory function was improved by xenon treatment. **(J)** Representative tracking results in the PFS and xenon treatment groups. ***P < 0.001, **P < 0.01 vs. the prolonged FS group. PFS, prolonged febrile seizure; PSD, power spectral density; Xe, xenon.

A shorter cumulative seizure duration (*P* < 0.001; Figure 7A) and fewer seizures (*P* < 0.001; Figure 7B) were observed in the xenon treatment group than in the group without xenon treatment (control; *n* = 18/time point) for pups. Synchronously, 8 out of 18 rats in the PFS group developed spontaneous seizures compared to only 2 out of 16 rats in xenon treatment group (Fisher’s exact test, *P* = 0.046). Furthermore, the elevated seizure frequency and duration of spontaneous seizures due to PFS were partly reversed by xenon inhalation (seizure frequency: 326.3 ± 100.9 vs. 9.5 ± 6.7; duration: 3189.4 ± 961.1 s vs. 99.8 ± 68.4 s; Figures 7C, D). The representative EEG and PSD analyses at 2 months are presented in Figure 7E, and similar attenuation of seizures at 2 months due to early xenon treatment were observed by EEGs analysis (Supplementary Figures 3A, B).

Learning and memory defects in adult rats were improved due to early xenon administration. The results demonstrated a longer target quadrant time (*P* < 0.001; Figure 7F), higher target zone frequency (Figure 7G), shorter opposite quadrant time (Figure 7H), and shorter latency to platform (*P* < 0.001; Figure 7I) in the xenon treatment group (*n* = 16) than in the control group (*n* = 18). Representative trajectory tracing diagrams of each group are shown in Figure 7J. These results show that xenon inhalation immediately after PFS can alleviate learning and memory deficits.

### 3.8. Xenon treatment ameliorated PFS-induced elevation of glutamate and oxidative stress

**FIGURE 8:**
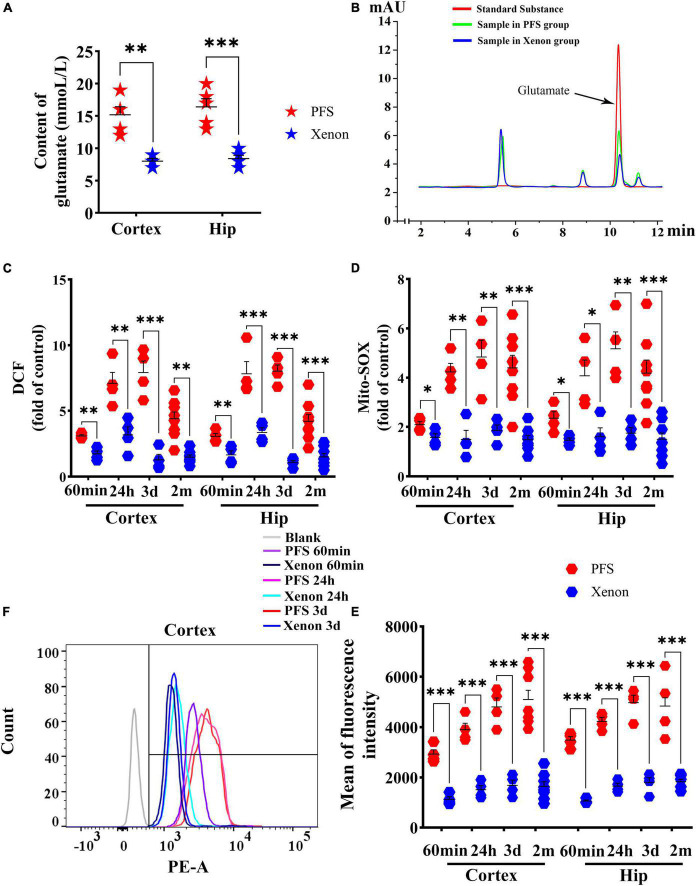
Xenon treatment ameliorated PFS-induced elevation in glutamate and oxidative stress. **(A)** Level of glutamate in the cortex and hippocampus after 60 min of xenon treatment (*n* = 5/group) and **(B)** representative high-performance liquid chromatography results of glutamate. Increased levels of DCF **(C)** and mito-SOX **(D)** detected by microplate reader were reduced owing to xenon treatment at 60 min (*n* = 4/group), 24 h (*n* = 4/group), 3 days (*n* = 4/group), and 2 months (*n* = 8/group). **(E)** Mean of fluorescence intensity of mito-SOX detected by flow cytometry at 60 min, 24 h, 3 days (*n* = 4/group), and 2 months (*n* = 8/group). **(F)** Representative flow cytometry results of mito-SOX. ***P < 0.001, **P < 0.01, and *P < 0.05 vs. the corresponding control groups. DCF, 2’,7’-dichloro-dihydro-fluorescein; Hip, hippocampus; PFS, prolonged febrile seizure.

After 60 min of xenon treatment, the elevation of glutamate was reversed almost to the control group level (hippocampus, *P* < 0.001; cortex, *P* = 0.001; Figure 8A). Representative HPLC results are shown in Figure 8B. Besides, DCF levels were significantly decreased in pups (60 min, cortex: *P* = 0.001, hippocampus: *P* = 0.001; 24 h, cortex, *P* = 0.003; hippocampus, *P* < 0.001; 3 days, cortex, *P* < 0.001; hippocampus, *P* < 0.001) and adults (cortex, *P* = 0.001; hippocampus, *P* = 0.001) by xenon treatment compared with those in the respective control groups (Figure 8C). Moreover, the mito-SOX levels were lower in the xenon treatment groups than in the PFS groups of both pups and adults (Figure 8D). In addition, the mean fluorescence intensity of mito-SOX detected by flow cytometry was lower in the xenon treatment group of pups (e.g., Day 3, cortex: *P* < 0.001) and adults (Day 60, *P* < 0.001) than that in the respective PFS groups (Figure 8E). Representative results of flow cytometry are shown in Figure 8F.

These results suggest that xenon treatment can significantly reduce overexcitation-induced oxidative stress in PFS rats.

### 3.9. Inhalation therapy of xenon reduced mitophagy levels

**FIGURE 9:**
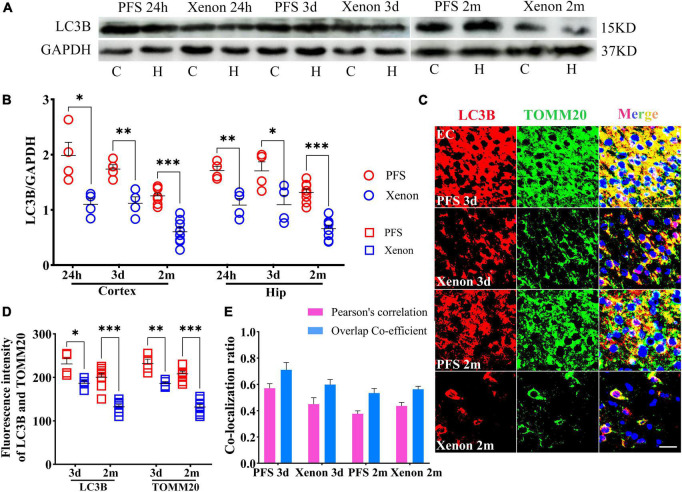
Inhalation therapy of xenon reduced mitophagy levels. **(A,B)** Elevated levels of LC3B were reversed by xenon therapy (24 h and 3 days, *n* = 4/group; 2 months, *n* = 8/group). **(C)** Representative images of immunoreactivity staining of LC3B (red) and TOMM20 (green) at 3 days (*n* = 4/group) and 2 months (*n* = 8/group), bar = 30 μm. DAPI, blue. **(D)** Mean fluorescence intensity of LC3B and TOMM20. **(E)** Pearson’s correlation and overlap coefficient of co-localization ratio. ***P < 0.001, **P < 0.01, and *P < 0.05 vs. the corresponding control groups. C, cortex; EC, entorhinal cortex; Hip/H, hippocampus; LC3B, microtubule-associated protein light chain 3B; PFS, prolonged febrile seizure; TOMM20, translocase of outer mitochondrial membrane 20.

The levels of LC3B were lower in the xenon inhalation therapy groups than in the control groups of pups and adults (Figures 9A, B). Immunohistochemical analyses revealed a similar trend of reduction in the levels of LC3B and TOMM20 (EC: LC3B, day 3, *P* = 0.013; day 60, *P* < 0.001; TOMM20, day 3, *P* = 0.005; day 60, *P* < 0.001; Figures 9C, D). The statistical results of colocalization are shown in Figure 9E. These results show that the increased level of autophagy (mainly mitophagy) induced by PFS can be reversed by xenon inhalation.

### 3.10. Xenon therapy alleviated the PFS-induced neural injury

**FIGURE 10:**
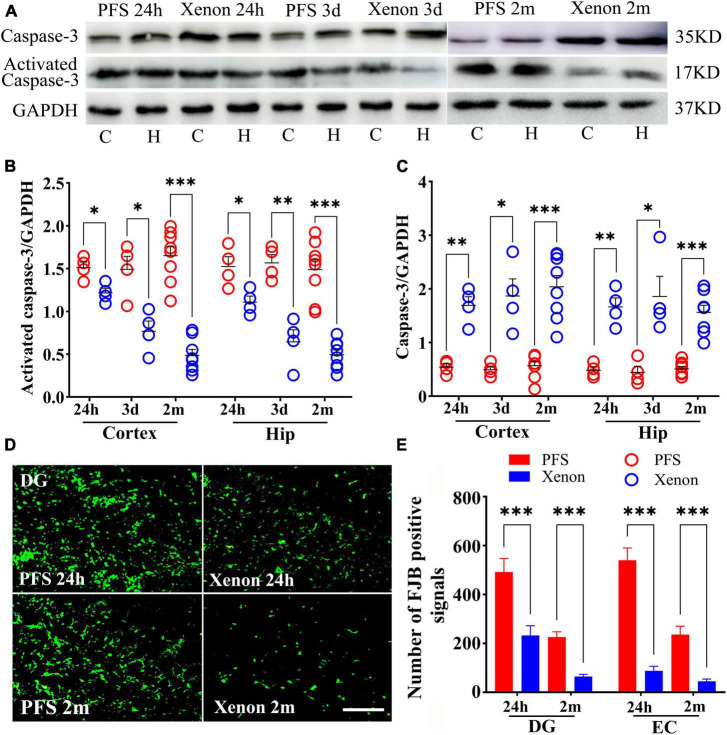
Xenon therapy alleviated the PFS-induced neural injury. **(A–C)** Increased levels of caspase-3 and decreased levels of activated caspase-3 owing to xenon treatment were observed at 24 h (*n* = 4/group), 3 days (*n* = 4/group), and 2 months (*n* = 8/group). **(D)** Representative FJB staining in the DG region at 24 h (*n* = 4/group) and 2 months (*n* = 8/group), bar = 50 μm. **(E)** Analysis of the number of FJB positive signals. ***P < 0.001, **P < 0.01, and *P < 0.05 vs. the PFS groups. C, cortex; DG, dentate gyrus; EC, entorhinal cortex; FJB, Fluoro-jade B; Hip/H, hippocampus; PFS, prolonged febrile seizure.

Xenon treatment decreased the levels of activated caspase-3 (24 h: cortex, *P* = 0.012; hippocampus, *P* = 0.013; day 3: cortex, *P* = 0.001; hippocampus, *P* < 0.001), and increased the levels of caspase-3 (24 h: cortex, *P* = 0.001; hippocampus, *P* = 0.001; Day 3: cortex, *P* = 0.006; hippocampus, *P* = 0.011; Figures 10A–C) in pups from the xenon treatment group compared with those in the control group. Similarly, in adult rats, decreased levels of activated caspase-3 and increased levels of caspase-3 were observed in the xenon treatment group compared with those in the control group (*n* = 8/group, Figures 10A–C).

Fluoro-Jade B staining demonstrated fewer positive signals in the xenon treatment group of pups at 24 h (DG, *P* < 0.001; EC, *P* < 0.001; Figures 10D, E) than in the PFS group of pups (*n* = 4/group). Similar attenuation was obtained in the xenon treatment group of adult rats at 2 months (*n* = 8/group; DG: *P* < 0.001; EC: *P* < 0.001; Figures 10D, E).

## 4. Discussion

Febrile seizures, which are induced by hyperpyrexia, constitute a common nervous disease that mainly occurs in children (Graves et al., 2012). They are classified into two types, according to the clinical characteristics: simple FS (duration of < 15 min) and complex FS (Subcommittee on Febrile Seizures and American Academy of Pediatrics, 2011; Thébault-Dagher et al., 2020). Although epidemiological studies have shown that simple FS have almost no significant influence on brain structure and cognition (Renda et al., 2020), a recent study confirmed that simple FS cause subtle changes in synaptic functions (Postnikova et al., 2021). Prolonged FS is considered to be harmful to the developing brain, may exacerbate the risk and severity of later recrudescence, and may even lead to intractable epilepsy (Camfield and Camfield, 2014).

Current drug treatments for FS rarely reduce the risk of epilepsy in later life and do not reduce neurological damage (Paul et al., 2015). Moreover, they negatively impact nervous system development in children (Moavero et al., 2017). Generally, AEDs are used in the clinical treatment of FS; not only do they rarely exhibit a neuroprotection effect on the brain, but they are also harmful to cerebral functional development in newborns (Paul et al., 2015; Moavero et al., 2017). Additionally, AEDs have many disadvantages. First, the mechanisms of action of several AEDs involve working through enhanced inhibition mediated by GABA receptor or transporter (Macdonald and Kelly, 1995). Research has demonstrated that the function of GABA-mediated inhibition in brain is not fully developed until 3 years old, and GABAergic even plays excitatory roles in several areas of the brain during the neonatal period (Dzhala et al., 2005). Therefore, AED treatment for FS might cause secondary noxious effects in the developing brain (Dzhala et al., 2005).

Xenon is an ideal anesthetic with excellent safety characteristics (Korsunsky, 2015). In the past 50 years, the safety of xenon has been strongly established in clinical settings (Wilhelm et al., 2002). Recently, xenon has attracted much attention for its powerful neuroprotective effects on neuronal injury in neurodegenerative diseases (Haseneder et al., 2009). According to previous experimental data, exposure of xenon within 2 h presented significant neuroprotective and anti-apoptosis effects without neurotoxicity in 7-day-old hypoxia-induced rats (Ma et al., 2006). In this study, we verified that xenon treatment immediately after PFS exhibits significant anti-seizure and neuroprotective roles, accompanied by a reduced risk of developing epilepsy.

Xenon plays an important role in balancing the flux of glutamate and inhibiting glutamate transport, which is the crux of acute neuronal injury, seizures, and epileptic development (Liu et al., 2010; Zhang et al., 2019a; Hanada, 2020; Maze and Laitio, 2020). Consequently, xenon treatment can significantly attenuate overexcitation-induced neuronal injury (Wilhelm et al., 2002; Dickinson et al., 2007; Harris et al., 2013; Campos-Pires et al., 2019) and rapidly terminate synchronous discharge induced by overexcitation in cortical neurons (Uchida et al., 2012). In this study, 44.4% of rats that suffered PFS in infancy subsequently developed spontaneous seizures in adulthood. However, after xenon treatment (applied only once after PFS), the number of rats that developed spontaneous seizures was reduced to 12.5% (Fisher’s exact test, *P* = 0.046). Moreover, even though the rats treated with xenon subsequently presented spontaneous seizures, the frequency and duration of seizures were milder than those in the PFS group (Supplementary Figure 4). A previous study reported that xenon does not show the psychotomimetic and neurotoxic properties that other anesthetics do (Shu et al., 2010). Besides, we have compared the effects of xenon (70%Xe/21%O_2_/9%N_2_) treatment in normal pups to evaluate the xenon influence on the brain development. Our results showed that there was no significant difference in neuronal damage between the control group (normal pups treated with 21%O_2_/79%N_2_) and xenon treatment group (Supplementary Figure 5). Compared with the negative effects of AEDs on nervous system development in children (Verrotti et al., 2012) and their barely significant role in preventing epileptogenesis induced by FS (Beghi, 2003), xenon mixture significantly attenuated seizures and alleviated the risk of PFS developing into epilepsy in later life.

Glutamate is an excitatory neurotransmitter in the brain (Hynd et al., 2004) that mediates neural hyperexcitation and plays a considerable role in neural injury, seizures, and the occurrence of epilepsy (Hanada, 2020). Previous studies have confirmed that elevated level of glutamate exerts a neurotoxic action by activating some glutamatergic receptors. N-methyl-D-aspartate (NMDA) receptors play a pivotal role in the regulation of excitability (Schwenkreis et al., 1999). Hyperactivation of NMDA receptors induced by elevated levels of glutamate can lead to neuroexcitotoxicity and cause neuronal injury (Maze and Laitio, 2020). However, elevated glutamate levels in the brain are shown to gradually return to normal levels several hours after a seizure (Ding et al., 1998; Mussulini et al., 2018), which might be associated with the closure of calcium channels by excessive depolarization during epilepsy (Ding et al., 1998; Steinlein, 2014). Our results indicate that the increased glutamate level was evidently reduced in the hippocampus and cortex at 6 h after PFS (Supplementary Figure 6).

Although the level of glutamate quickly decreased because of endogenous antiepileptic mechanisms, glutamate-induced neurotoxicity in the brain was considerable (Mussulini et al., 2018). Glutamate-mediated excitotoxicity due to combination with NMDA receptor in the early period could increase calcium influx and oxidative stress (Yu et al., 2016) and then aggravate mitophagy and cell death (Gao, 2019), which are important for the formation and development of the excitatory network of epilepsy at a later date (Yu et al., 2016; Wu et al., 2019; Yang et al., 2020). Additionally, the overexcitation-induced injuries could in turn promote the excessive production of ROS in later life (Puttachary et al., 2015). Therefore, the increased glutamate-mediated excitotoxicity may trigger an increase of oxidative stress and epileptogenesis. Our results confirmed that in the early period after PFS, the levels of glutamate and oxidative stress were synchronously increased. Additionally, though the glutamate levels were elevated transiently after PFS, the oxidative stress levels were always higher than those in the controls, even in adulthood.

Xenon can decrease excitotoxicity by decreasing the glutamate level in earlier periods and exerting neuroprotective effects (Weigt et al., 2009). Kubota et al. (2020) found that xenon could significantly inhibit the glutamatergic responses in the hippocampal region of brain, reducing the uptake of glutamate. Besides, some evidence suggests that xenon inhibits transmission and uptake of glutamate as well as further inhibit NMDA receptors through binding at the interface of NR1 and NR2 subunits (Dickinson et al., 2007; Liu et al., 2010; Zhang et al., 2021). Recently, TREK-1 gene was found to be activated by xenon, which is considered to be associated with the activation of Ca^2+^ channels, reduction of glutamate release and inhibition of excitotoxicity (Zhao et al., 2018). A steadily accumulating body of evidence shows that xenon can decrease the level of glutamate, antagonize NMDA receptors, and attenuate neuroexcitotoxicity-induced oxidative stress (Lavaur et al., 2016; Zhang et al., 2021). Our results confirmed that one round of xenon treatment immediately after PFS significantly reduced the increased glutamate levels in the early period, accompanied by an attenuation of the oxidative stress and neuronal injury even into adulthood. These results suggest that reducing the increased glutamate levels during early PFS may contribute to the protection effect provided by xenon.

Mitochondria are the main site of ROS generation and thus play a vital role in balancing cell life and death (Abate et al., 2020). Mitophagy plays a key role in mitochondrial quality control and homeostasis and contributes to cell function and integrity (Montava-Garriga and Ganley, 2020). However, mitophagy may be a “double-edged sword,” playing different roles under different physiological and pathological conditions (Pickles et al., 2018; Montava-Garriga and Ganley, 2020). Mitochondria are sensitive to the level of oxidative stress (Cheng et al., 2021), and excessive ROS results in mitochondrial defects, aggravating mitophagy (Puttachary et al., 2015). Conversely, immoderate mitophagy could change transient Ca^2+^ states and promote the production of ROS, resulting in epileptogenesis (Han et al., 2011). The present study confirmed that the increased levels of oxidative stress and mitophagy induced by PFS were partly reversed in xenon-treated rats and were accompanied by reversed glutamate levels. These results may be associated with xenon treatment reversing the increased levels of glutamate and oxidative stress, alleviating mitochondrial defects, and then reducing the level of mitophagy. Whether the reduced mitophagy is a consequence of xenon protection or only the concomitant result because xenon treatment attenuated oxidative stress-induced neuronal injury needs further verification.

Cysteine proteases (caspases) are a family of central regulators of apoptosis in cells (Henshall and Simon, 2005; D’Amelio et al., 2010). The activation of caspases is an important biochemical hallmark of apoptosis (Henshall and Simon, 2005; D’Amelio et al., 2010; Choudhary et al., 2015). Caspase-3 has been identified as a crucial mediator of apoptosis in neurons (D’Amelio et al., 2010), and reduced caspase-3 activation would lead to the inhibition of apoptosis (D’Amelio et al., 2010; Choudhary et al., 2015). Additionally, previous research has demonstrated the cascade signaling of apoptosis induced by caspase-3 during oxidative stress injury (Kim et al., 2020; Vega-García et al., 2021). Glutamate-mediated excitotoxicity could cause the excessive production of ROS and neuronal injury, triggering the caspase 3-induced apoptosis signaling pathway (Levite, 2014; Zhao et al., 2021), even leading to cell death (Zhang et al., 2021); this process is considered to be closely related to epileptic development (Yang et al., 2016). According to the previous reports, xenon could attenuate glutamate accumulation (Roostan and Frishman, 2018), reduce the level of oxidative stress (Lavau et al., 2017), and alleviate cell apoptosis (Gill and Pickering, 2021). Therefore, it is reasonable to consider that the anti-apoptosis effects may participate in the neuronal protection of xenon through decreasing the elevated oxidative stress level.

The limbic system and hippocampal regions are crucial for cognitive function and epileptic neural networks (Krzywkowski et al., 1994). As an important “gatekeeper” of memorizing information, the EC is responsible for processing memory information and transmitting it to the hippocampus for integration (Coutureau and Di Scala, 2009). The hippocampus is central to the neural memory system and provides the spatial framework within which events are located and interrelated (Senzai, 2019; Treder et al., 2021). In this process, the dentate gyrus (DG) plays a key role in information recognition in the hippocampus, which is known as a “cognitive map” (Brandeis et al., 1989; Hainmueller and Bartos, 2020). Meanwhile, the connectivity integrity of medial temporal lobe has an indispensable relationship with epilepsy, especially TLE (Tramoni-Negre et al., 2017; Berron et al., 2020). Moreover, PFS can lead to hippocampal damage, especially in the DG, a crucial region of the hippocampal network (Hoogland et al., 2022), and results in long-term effects on the excitability of the hippocampus (Acharya et al., 2022), which might also be one of the vital causes of the development of TLE later in life. Notably, both neuronal injuries and cognitive defects were significantly diminished by xenon treatment immediately after PFS, with attenuated seizures and reduced risk of epilepsy. These findings indicate that cognitive defects and epileptic development after PFS might be related to neural injuries in neonatal period, which causes the neural network to be exceptionally excited, and increases the likelihood of epilepsy and cognitive defects later in life (Shetty, 2014; Yu et al., 2016). Xenon improved cognitive function, decreased the severity of seizures, and reduced epileptic risk in adulthood, which might be closely related to alleviated nervous damage during the neonatal period.

However, the rarity of xenon in the atmosphere leads to an expensive market price for this product and reduce the attractiveness of its use in clinical treatments (Neice and Zornow, 2016). Therefore, creative technologies are needed to develop solutions for decreasing the costs and improving the recycling capacity of this product. It has been found that xenon may be recycled because it was shown to be exhaled directly from the body without being metabolized (Delhaye et al., 2010), which has provided a theoretical basis for the sustainable utilization of xenon. Recently, a closed rebreathing system was developed, which promotes the widespread application of xenon (Delhaye et al., 2010). Besides, the use of circulating equipment is conducive to reducing costs and promoting clinical popularization (Hanne et al., 2001). Combined with its satisfactory safety, our study suggests that xenon might be safe and effective in treating FS and preventing epileptic development later in life.

Febrile seizures not only significantly increases seizure susceptibility but also genetic predisposition in offspring (Huang and Chang, 2009; Wu et al., 2017). Family investigations have revealed that this disease is inherited by autosomal dominant inheritance (Kira et al., 2010). A recent study identified several genetic susceptibility genes, including FEBs and SCNs (Nakayama, 2009). Therefore, it would be worthwhile to explore whether xenon treatment could reduce epileptic susceptibility in the offspring of PFS models.

In summary, our study provides compelling evidence for the effectiveness of xenon treatment not only for the treatment of FS (especially PFS) but also for decreasing the risk of epileptic development in later life. As a safe, effective, and environmentally friendly gas, xenon might be a prospective new treatment strategy for FS in the future.

## Data availability statement

The original contributions presented in this study are included in this article/Supplementary material, further inquiries can be directed to the corresponding authors.

## Ethics statement

The animal study was approved by the Binzhou Medical University Experimental Animals Committee (approval no. 2020002). The study was conducted in accordance with the local legislation and institutional requirements.

## Author contributions

HS, YZ, YC, and YJZ: conception and design of study. HL: HPLC detection. YC, YZ, YY, HL, WZ, ZF, LZ, XG, YJZ, and HS: data collection and analysis. YC and YJZ: methodology and writing—original draft. YJZ and YY: data curation. HS and LZ: resources. HS, YZ, and YC: writing—review and editing. All authors contributed to the article and approved the submitted version.
