# Supplementary material for: Xenon inhalation attenuates neuronal injury and prevents epilepsy in febrile seizure Sprague-Dawley pups
Source: Front Cell Neurosci. 2023 Aug 14;17:1155303. doi: 10.3389/fncel.2023.1155303 (PMC10461106; doi:10.3389/fncel.2023.1155303)
Supplement: Supplementary file 1 [file Table_1.DOCX]

The raw data of this manuscript has been uploaded: https://www.jianguoyun.com/p/DRxNcewQkKr7ChjyuvMEIAA
