# Supplementary material for: Xenon inhalation attenuates neuronal injury and prevents epilepsy in febrile seizure Sprague-Dawley pups
Source: Front Cell Neurosci. 2023 Aug 14;17:1155303. doi: 10.3389/fncel.2023.1155303 (PMC10461106; doi:10.3389/fncel.2023.1155303)
Supplement: Supplementary file 2 [file Data_Sheet_1.docx]

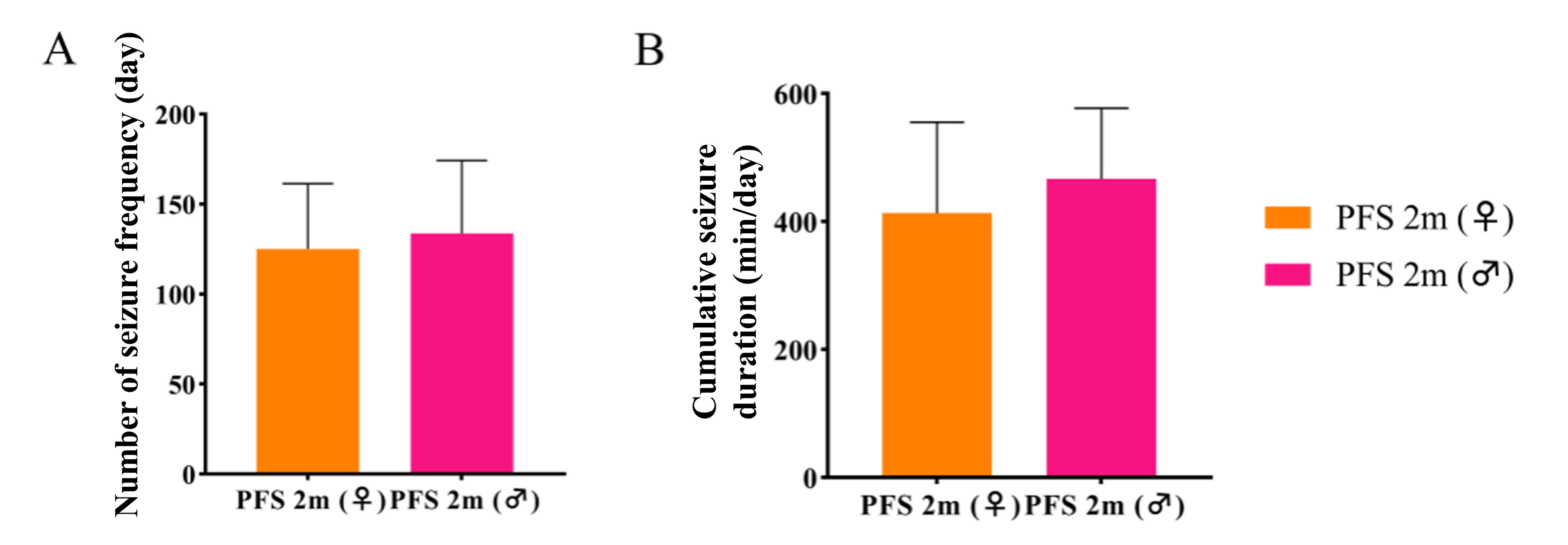


**Supplementary Figure 1.** The analysis of EEGs in PFS rats at 2 months. (A) Number of seizure frequency and (B) cumulative seizure duration (female, n = 16; male, n = 20). Mean ± SEM were presented. PFS, prolonged febrile seizure.


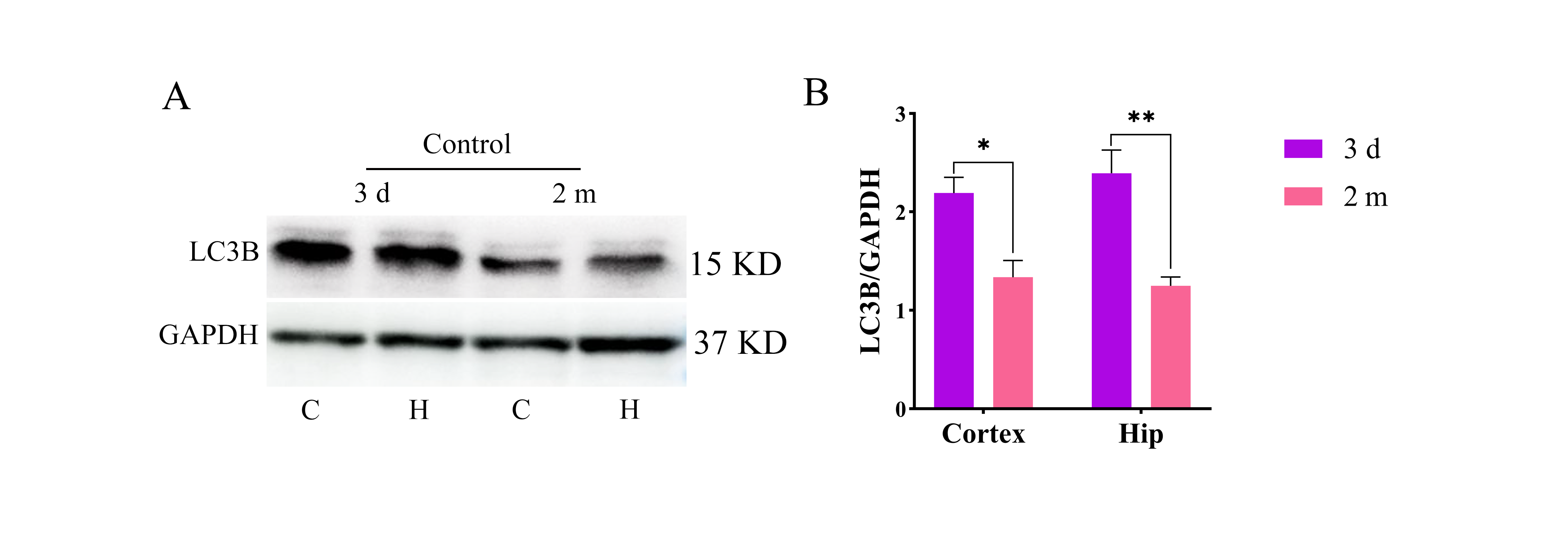


**Supplementary Figure 2.** (A, B) The levels of LC3B in normal rats at 3 days and 2 months (n = 4/group). Mean ± SEM were presented. ***P < 0.01, *P < 0.05 vs* each other. C, cortex; H, hippocampus; LC3B, microtubule-associated protein light chain 3B.


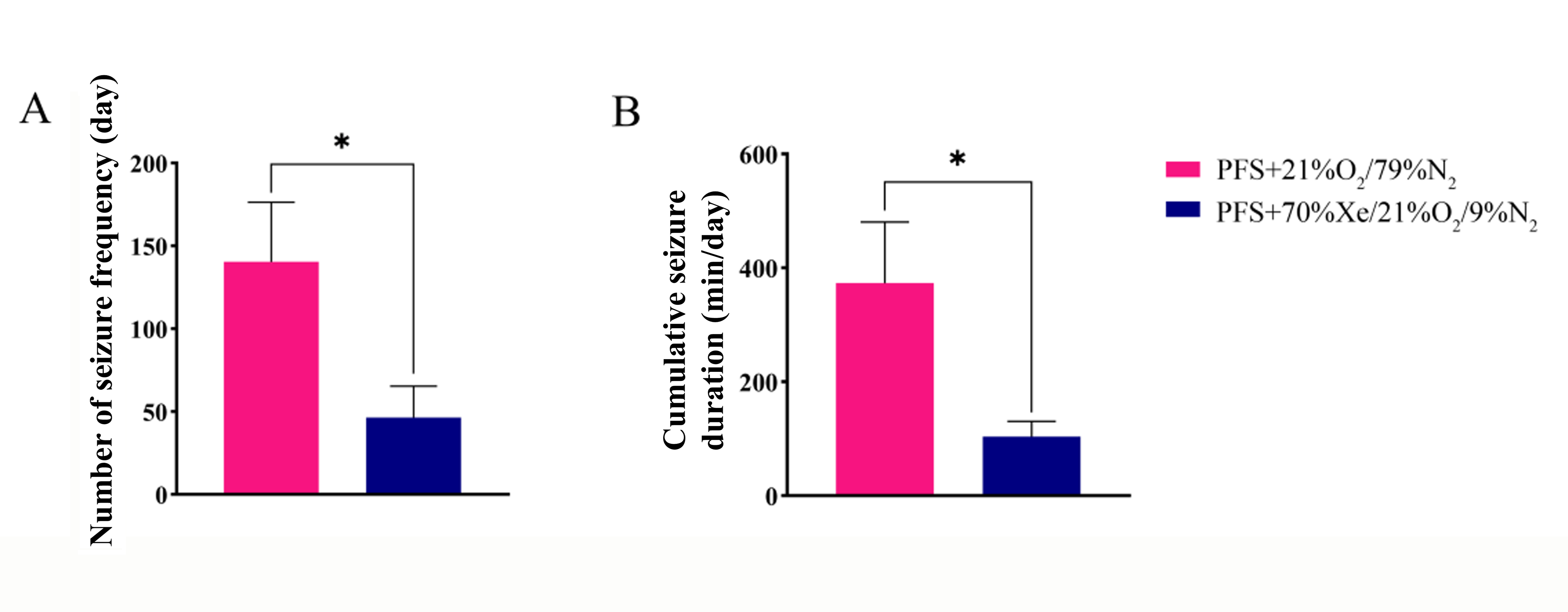


**Supplementary Figure 3.** The EEGs analysis in each group at 2 months. (A) Number of seizure frequency and (B) cumulative seizure duration. Mean ± SEM were presented. **P < 0.05 vs* the control group (n = 18). PFS, prolonged febrile seizure.


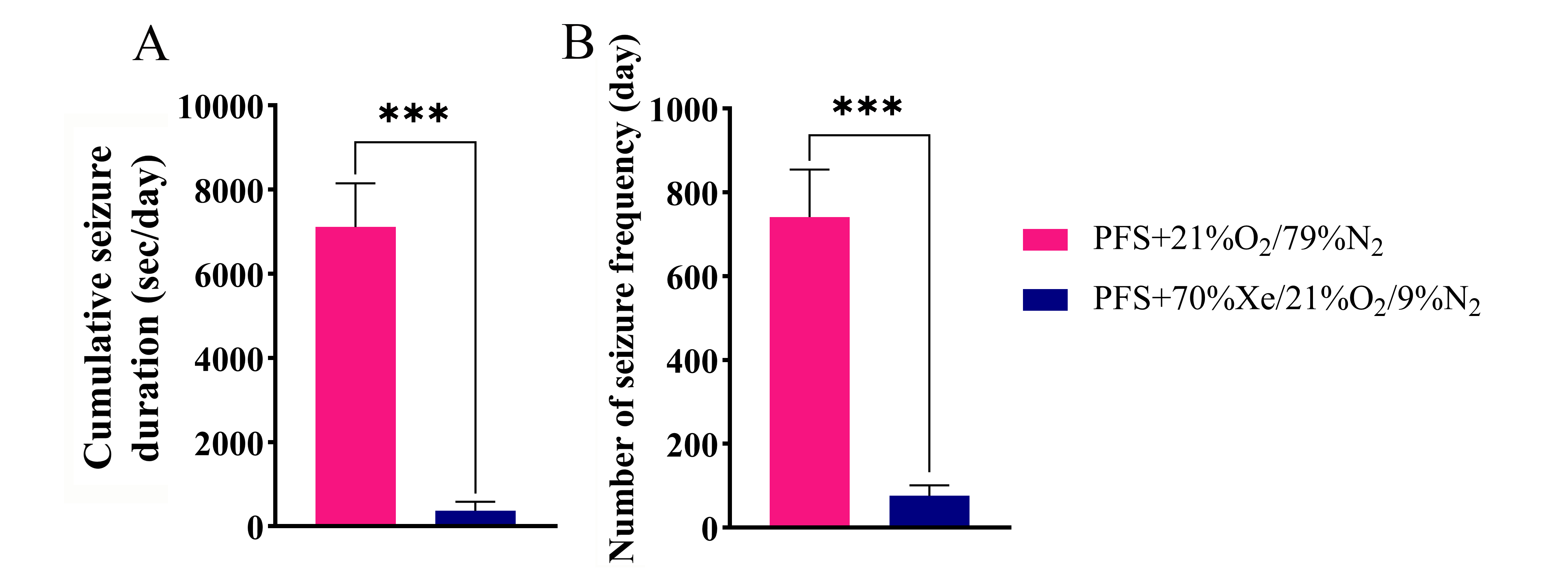


**Supplementary Figure 4.** The behavioral analysis of spontaneous seizures rats in xenon group and PFS group at 2 months. (A) Cumulative seizure duration and (B) frequency of seizures. Mean ± SEM were presented. **P < 0.05 vs* the corresponding control group. PFS, prolonged febrile seizure.


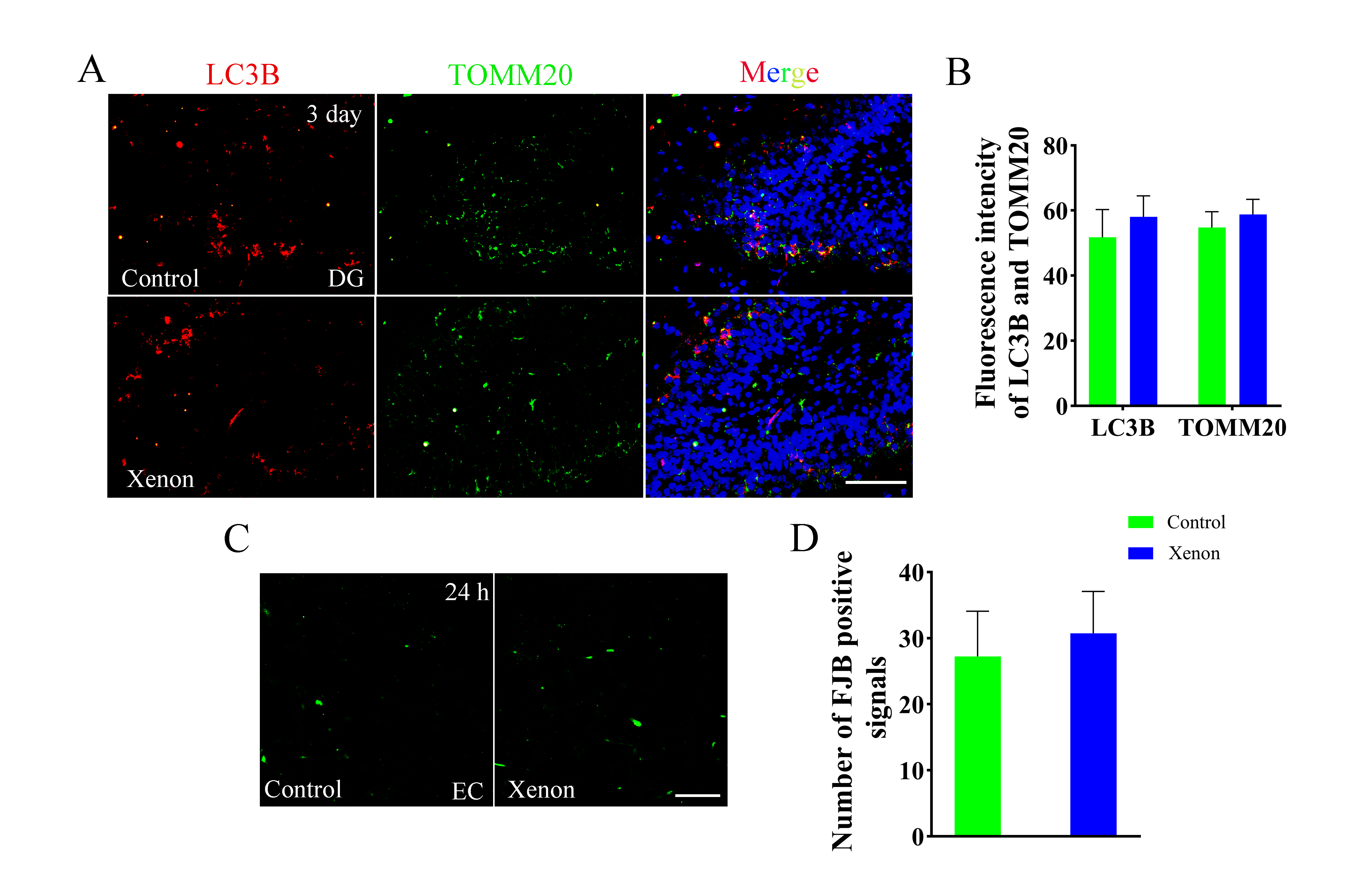


**Supplementary Figure 5.** The analysis of mitophagy and neuronal injury levels between control and xenon treatment groups.

(A, B) The mean fluorescence intensity of LC3B and TOMM20. Bar = 100 μm. (C, D) The number of FJB positive signals analysis. Bar = 30 μm, n = 4/group. Mean ± SEM were presented.


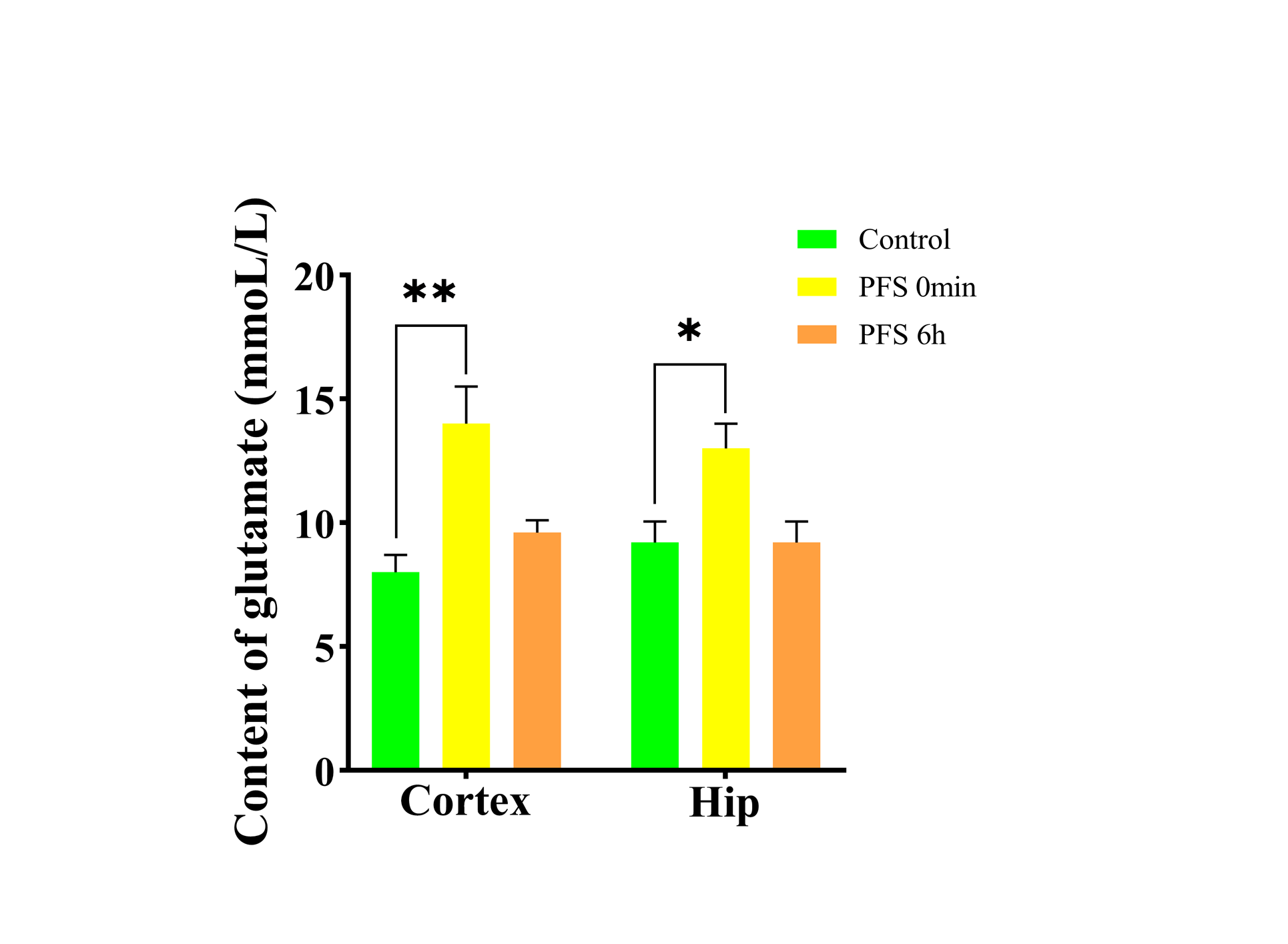


**Supplementary Figure 6.** The level of glutamate in each group at 0 min and 6 hours after PFS. Mean ± SEM were presented. ***P < 0.01, *P < 0.05 vs* the control group (n = 5/group). PFS, prolonged febrile seizure; Hip, hippocampus.
