# Supplementary figures and images for: Xenon inhalation attenuates neuronal injury and prevents epilepsy in febrile seizure Sprague-Dawley pups
Source: Front Cell Neurosci. 2023 Aug 14;17:1155303. doi: 10.3389/fncel.2023.1155303 (PMC10461106; doi:10.3389/fncel.2023.1155303)

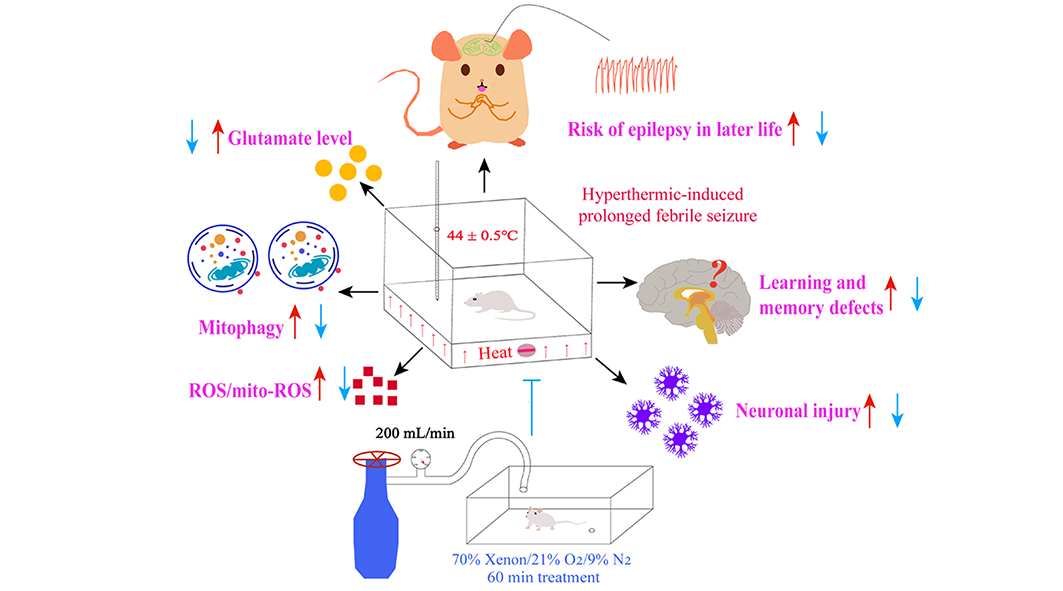

Supplement: Supplementary file 3 [file Image_1.TIF]
